# Comparison of Two Techniques for the Detection of Flea Faeces in Canine and Feline Coat Brushings

**DOI:** 10.1155/2014/292085

**Published:** 2014-10-21

**Authors:** Marie-Christine Cadiergues, Caroline Cabaret-Mandin, Chloé Solatges

**Affiliations:** Dermatology Unit, Department of Clinical Sciences, Institut National Polytechnique-Ecole Nationale Vétérinaire de Toulouse (INP-ENVT), 23 Chemin des Capelles, BP 87614, 31076 Toulouse Cedex 3, France

## Abstract

Flea infestation is diagnosed after the detection of either adult parasites or flea faeces in the fur. The latter is generally tested with the wet blotting paper technique (WBPT). However, microscopical examination (MT) of the coat brushing material is sometimes suggested as an alternative. This study aimed to compare the efficiency of the two techniques. In dogs, the entire body was hand-brushed and cats were combed. One half of the collected material was mounted in liquid paraffin on a glass slide and examined microscopically at low magnification. The second half was placed on a blotting paper and sterile water was added. After drying, reddish aureoles were counted. 255 animals (158 dogs and 97 cats) were included. 119 (47%) and 94 (37%) samples were revealed to be positive with WBPT and MT, respectively. 13 cases (5%) were positive with MT only and 38 cases (15%) were positive with WBPT only. 81 cases (32%) were positive and 123 (48%) were negative with both techniques. More positive cases were detected by WBPT than MT (*P* < 0.001). Amongst the 51 samples which were found positive with a sole technique, infestation was considered low in 43 cases and WBPT detected significantly more positive samples (31) than MT (12), *P* < 0.01.

## 1. Introduction

Fleas represent the most important ectoparasites in companion animals. More than 2000 species and subspecies are identified throughout the world. Nevertheless, very few species are encountered on dogs and cats and, amongst these,* Ctenocephalides felis felis* is far the most common [1]. Fleas are primarily a nuisance for dogs and cats due to mechanical irritation and, in heavily infested animals, possible anemia [2]. Dermatological signs become evident when animals develop flea allergy dermatitis (FAD). Flea infestations can be diagnosed based on clinical signs and observation of fleas or flea dirt (dried flea feces). A flea comb may be used to collect material from the coat for gross examination. If fleas are present in large numbers, they should be seen. However, in mild or low infestations, combing is poorly sensitive [3, 4]. Proof of flea exposure may also be obtained with evidence of flea faeces in the coat. Fleas produce about 0.77 mg of feces per day [5]. Two types of flea feces are produced: spherules and coils [6]. In order to detect flea dirt, it is generally recommended to use the wet blotting paper technique. Suspected flea dirt is placed on moistened white paper and will dissolve to an orange-red stain as it contains partially digested haemoglobin [7]. However, in a pruritic animal, it may be difficult to distinguish between flea dirt and haemorrhagic crusts. Alternatively, coat brushing material can be examined microscopically in order to detect characteristic dark red comma-shaped and/or round elements [8]. The purpose of this study was to compare the efficiency of the two methods in order to give the best advice to veterinarians in the field.

## 2. Materials and Methods

### 2.1. Animals

The study was conducted at the Small Animal Hospital of the Toulouse Veterinary School, Toulouse, France Owner consent was obtained for each animal before sampling. Dogs and cats were excluded if they were unwell, were difficult to manipulate, or had extensive skin lesions. They were also excluded if they had flea infestation detectable with a visual coat examination. Collected data included breed, age, gender, frequency, and date of the last application of an ectoparasiticide and/or a shampoo.

### 2.2. Collection of the Coat Brushing Material

#### 2.2.1. Dogs

The entire body of the animal was vigorously brushed with the fingertips either directly on the surface of a cleaned examination table for large dogs or on a piece of A4 paper for small dogs.

#### 2.2.2. Cats

Each cat was combed with an extra-fine flea comb (11.4 teeth/cm, Mikki http://www.petmeds.fr/). Additionally, debris were shaken off the travel basket if available and thereafter collected.

### 2.3. Preparation of the Samples

Hair was removed and the amount of material was divided into two equal parts. One half was placed on a glass slide (76 × 26 mm, Menzel-Gläser, Braunschweig, Germany) and mounted in liquid paraffin under a 22 × 22 mm coverslip (Menzel-Gläser, Braunschweig, Germany). The second half was placed on a folded blotting paper measuring 37 × 100 mm (Labo-Moderne, Paris, France—ref LMRSL). Both were labelled and stored until examined within the next 10 hours. All the procedure was performed by the same person (CCM).

### 2.4. Examination of the Samples

Two investigators (MCC and CS) were involved in the examination phase. They were blinded to the animal data. To avoid bias, the two investigators alternated between the two techniques every 20 samples. Before specific examination, each sample was examined grossly as positive or negative for the presence of flea faeces.

#### 2.4.1. Wet Blotting Paper Technique (WBPT)

Based on unpublished preliminary studies, 3 drops of sterile water (10 mL vials, eau pour préparation injectable ProAmp, Aguettant, Lyon, France) were added after having manually scattered the material. The paper was left to dry for 10 minutes. If needed, the remaining debris were removed by a little bump on the flip side of the paper. Finally, areas with reddish aureoles were counted.

#### 2.4.2. Microscope Technique (MT)

The area defined by the coverslip surface was examined at low magnification (×40), starting at one corner and progressing systematically by successive horizontal rows. The light intensity was moderate and the condenser lowered. Characteristic dark red comma-shaped and/or round elements were counted.

The severity of the infestation was determined semiquantitatively according to the numbers of fecal elements. Infestation was absent when no fecal element was detected; infestation was low when one or two fecal elements of small size were observed; infestation was moderate if three to nine fecal elements were present and infestation was severe with ten or more fecal elements of large size.

### 2.5. Statistical Analysis

Data were entered into an Excel spreadsheet and statistical tests were performed in WinSTAT for Microsoft Excel (version 2012-1, copyright © 2012 Robert K. Fitch). To determine significance of tested parameters, chi-square test and contingence tables were used. Significance was set at 0.05.

## 3. Results

Two hundred and fifty-five animals were included and comprised 158 dogs and 97 cats. One hundred and nineteen (47%) and 94 (37%) samples were revealed to be positive with WBPT and MT, respectively. Thirteen cases (5%) were positive with MT only and thirty-eight cases (15%) were positive with WBPT only. Eighty-one cases (32%) were positive and 123 (48%) were negative with both techniques. More positive cases were detected by WBPT than by MT (*P* < 0.001). Amongst the 51 samples which were found positive with a sole technique, infestation was considered low in 43 cases; the remaining 8 samples were moderately infested. In the 43 cases detected with a low infestation by only one technique, WBPT detected significantly more positive samples (31) than MT (12), *P* < 0.01. Amongst the 81 samples which were found positive with both techniques, the estimation of the infestation severity was similar between the two techniques in 41 cases (50.1%), there was a one-level discrepancy in 32 cases (39.5%), and there was a two-level discrepancy in 6 cases (7.4%). Infestation was considered high by at least one technique in 43 cases and moderate in 37 cases. When comparing the performances of the two investigators, no differences were detected (*P* = 0.05).

Prior to the examination of each sample, the investigators observed grossly the collected material. Thirty-eight (15%) and 22 (8.6%) samples initially found negative with a naked eye proved positive with WBPT and MT, respectively. Conversely, 19 (7.6%) and 14 (5.5%) samples initially found positive with the naked eye did not reveal any fecal material with WBPT. Overall, gross examination overestimated the presence of fecal material in 6.5% of the samples and underestimated flea faeces in 11.7% of the cases.

Details for each species are presented in Table 1. Coat brushing collection from cats held significantly more positive samples than those collected from dogs (*P* < 0.001).

Thirty-six dogs and 36 cats were never treated against fleas, 76 dogs and 40 cats were treated irregularly, and 46 dogs and 21 cats were treated regularly and up to date (Table 2). Cats were treated significantly less regularly than dogs (*P* < 0.05). However, this difference did not influence the result of the coat brushing examination (*P* = 0.05).

Thirty-seven dogs and two cats had been shampooed in the two weeks prior to the inclusion. In dogs, results did not differ significantly between dogs which had been shampooed recently and the rest of the population. There were too few shampooed cats to allow a comparison.

## 4. Discussion

In 1994, Heckenberg and colleagues [9] showed that comb-counting recovered significantly (*P* ≤ 0.05) more fleas than did thumb-counting. On dogs given 50 and 100 fleas, comb-counting for 8 min gave mean percentage recoveries of 67.6% and 75.4%, respectively, whereas thumb-counting (mean time 3.2 min) found means of 8.8% and 7.7%, respectively. In 1995, Gregory and colleagues [10] specified in a comparable study that the differences between the two techniques were independent of time. However, these two studies were conducted on Beagle dogs, a breed easy to comb. In the field, most of the dogs are not easy to comb, due to the length or the texture of the hair coat. Therefore, thumb-counting or gross inspection done by the veterinarian during a clinical examination can be insufficient and flea infestation may be overlooked. Overlooking flea infestation (also with sole confidence in an up-to-date treatment) may lead to misdiagnosis of pulicosis or FAD; hence, it is a necessity to look for evidence of flea faeces as a proof of flea exposure. The aim of this prospective, double-blind study was to compare WBPT, usually recommended with MT, less commonly used, in the detection of flea fecal material in dogs and cats. It was expected that the microscopic observation would be more efficient than flea dirt observation on a wet blotting paper, particularly as it could detect very small pieces of faeces.

In this study, WBPT appears significantly more efficient than MT, mainly for infestation of low severity. Unfortunately, sensitivity and specificity of each technique could not be calculated since there is currently no reference technique to detect flea infestation in pets. The use of oral nitenpyram, a fast-acting insecticidal product, could be an option, provided that fleas are not disturbed and can take a blood meal [11].

Specificity of WBPT remains questionable when the sample is taken from a pruritic animal as hemorrhagic crusts can be mistaken for flea faeces based on the wet paper discoloration. As the same material could not be utilised successively for both techniques for technical reasons (need of liquid paraffin in MT and water in WBPT), the amount of collected material was divided into two equal parts. This, inevitably, reduces the chances to observe fecal material for each technique, particularly in low infested samples.

This study also shows that gross examination of the samples is not sufficient on its own to diagnose flea infestation, as it may overestimate or underestimate flea infestation. The overestimation can occur with dark-colored debris which can be mistaken for fecal material; conversely, when fecal elements are of small size, they can be underlooked.

Cats were more often infested than dogs (60% and 48%, resp.) and more severely infested. The difference might be explained by different collection methods: all cats were combed and material was also collected from their travel basket whereas dogs were vigorously brushed with the fingertips. The choice of a different methodological approach was guided by the field: most cats would not tolerate vigorous fingertips brushing and many canine breeds have a hair coat which is not compatible with an extra-fine flea comb. However, as the study showed, antiflea treatment in cats is less common than in dogs, which could support a higher level of infestation in cats. Flea infestation in cats is frequently overlooked and possibly denied [12]. In a study conducted in 2007 in the UK, 48% of the owners whose pets had signs of an active flea infestation were unaware that their pets had fleas [13]. Amongst the pets that were regularly treated against fleas and with up-to-date treatment, 11.4% of dogs and 18.6% of cats had evidence of flea dirt in their coat brushing. Pet owners, as well as some practitioners, often bring up resistance to flea products as soon as there is evidence of fleas on their recently treated pet [14]. Most of time, the treatment failure can be explained by a treatment deficiency as true resistance is rare.

In conclusion, this study has shown that WBPT was more efficient than MT in detecting the presence of flea faeces in the coat brushing material, especially in low level infestations. In moderate or high level infestations, a sole technique would be sufficient, whereas in pets with very few fleas, our recommendation would be to combine the two techniques. Practitioners should not assess the presence of fleas only on gross examination of the coat brushing. This study also confirmed the higher level of flea infestation in cats compared to dogs as well as the presence of fleas in pets supposedly correctly treated. Further studies are required to determine sensitivity and specificity of both techniques.

## Figures and Tables

**Table 1:** Results of the examination of the coat brushing material using two techniques in dogs and cats.

	Microscope technique		Total
	Positive	Negative	
Blotting paper technique			
Dogs			
Positive	26	30	56
Negative	7	95	102
Cats			
Positive	55	8	63
Negative	6	28	34

Total	94	161	255

**Table 2:** Results of the examination of the coat brushing material in dogs and cats according to the flea treatment status.

	Flea treatment			Total
	Regular	Occasional	Absent	
Dogs				
Negative^1^	28	49	18	95
Positive^2^	18	27	18	63
Cats				
Negative^1^	3	16	9	28
Positive^2^	18	24	27	69

Total	67	116	72	255

^1^Negative result with WBPT and MT; ^2^positive result with at least one technique (WBPT or MT).
